# Comparing Outcomes of a Digital Commercial Weight Loss Program in Adult Cancer Survivors and Matched Controls with Overweight or Obesity: Retrospective Analysis

**DOI:** 10.3390/nu13092908

**Published:** 2021-08-24

**Authors:** Christine N. May, Annabell Suh Ho, Qiuchen Yang, Meaghan McCallum, Neil M. Iyengar, Amy Comander, Ellen Siobhan Mitchell, Andreas Michaelides

**Affiliations:** 1 Academic Research, Noom Inc., 229 W. 28th St., New York, NY 10001, USA; christinem@noom.com (C.N.M.); annabell@noom.com (A.S.H.); qy2182@caa.columbia.edu (Q.Y.); meaghanm@noom.com (M.M.); andreas@noom.com (A.M.); 2 Memorial Sloan Kettering Cancer Center, 300 East 66th St., New York, NY 10065, USA; iyengarn@mskcc.org; 3 Massachusetts General Hospital Cancer Center, Harvard Medical School, 55 Fruit St., Boston, MA 02114, USA; acomander@mgh.harvard.edu

**Keywords:** weight loss, obesity, cancer survivors, retrospective study

## Abstract

Maintaining a healthy weight is beneficial for cancer survivors. However, weight loss program effectiveness studies have primarily been in highly controlled settings. This is a retrospective study exploring real-world outcomes (weight loss and program engagement) after use of a digital commercial weight loss program (Noom) in cancer survivors and matched controls. All participants had voluntarily self-enrolled in Noom. Weight and engagement data were extracted from the program. Cancer-related quality of life was secondarily assessed in a one-time cross-sectional survey for survivors. Controls were a sample of Noom users with overweight/obesity who had no history of cancer but 0–1 chronic conditions. Primary outcomes were weight change at 16 weeks and program engagement over 16 weeks. Engagement included frequency of weight, food, and physical activity logging, as well as number of coach messages. Multiple regression controlling for baseline age, gender, engagement, and BMI showed that survivors lost less weight than controls (B = −2.40, s.e. = 0.97, *p* = 0.01). Survivors also weighed in less (survivors: 5.4 [2.3]; controls: 5.7 [2.1], *p* = 0.01) and exercised less (survivors: 1.8 [3.2]; controls: 3.2 [4.1], *p* < 0.001) than controls. However, survivors sent more coach messages (survivors: 2.1 [2.4]; controls: 1.7 [2.0], *p* < 0.001). Despite controls losing more weight than cancer survivors (−7.0 kg vs. −5.3 kg), survivors lost significant weight in 4 months (M = −6.2%). Cancer survivors can have success on digital commercial programs available outside of a clinical trial. However, they may require additional support to engage in weight management behaviors.

## 1. Introduction

The number of cancer survivors within the United States continues to increase rapidly as treatments improve and screening efforts expand. Over the next twenty years, the population of cancer survivors is expected to increase nearly two-fold, reaching 26.1 million individuals [1]. Given this projection, anticipating the complex health needs of cancer survivors represents a major public health concern [2]. Maintenance of a healthy body mass index (BMI) is one modifiable risk factor that has been associated with decreased risk for recurrence and mortality for survivors of certain types of cancers [3]. Current guidelines from the American Cancer Society and American Society of Clinical Oncology recommend maintaining a healthy weight after cancer treatment, but cancer survivors receive insufficient guidance on weight management from health providers [3,4]. Cancer survivors may try to manage their weight on their own or through commercial programs outside of a clinical trial. These self-management methods are estimated to be the most common weight management strategies [5].

In particular, commercial digital programs are rapidly proliferating due to widespread smartphone access in various geographical areas and among diverse populations [6,7]. These digital commercial programs raise important new empirical questions for cancer survivors. For instance, individuals use these programs in the comfort of their own home, which means they self-manage their participation in the absence of monitoring requirements in research protocols or in-person clinical sessions [5,8,9]. However, extant knowledge of weight management outcomes and behaviors is almost exclusively derived from formal study, clinical, or in-person settings. For the increasing number of cancer survivors who use digital commercial programs, the extent of their outcomes and behaviors in their real-world use of the program is entirely unknown.

Therefore, we conducted a retrospective analysis of weight loss and engagement in a commercially available digital program among self-enrolled cancer survivors with overweight or obesity compared with a group of matched controls. This question is particularly important for cancer survivors, who may have disease-related barriers to participation. Cancer survivors face post-treatment challenges, such as cancer-related fatigue and lack of energy, side effects, new health conditions, and physical limitations [10]. Survivors have also reported difficulty in self-sustaining weight-relevant behavioral changes [11]. In addition, weight gain is more common in breast cancer survivors than in non-cancer patients [12]. Moreover, commercially available weight loss programs are not typically designed specifically for cancer survivors. Thus, we hypothesized that matched controls would have greater weight loss and engagement than cancer survivors. We also conducted a subgroup analysis of breast cancer survivors since this was the most commonly reported cancer type and because obesity is associated with increased risk of breast cancer recurrence [13]. An additional aim of the study was to descriptively report survivors’ cancer-related quality of life (QoL) after using this commercial digital weight loss program.

## 2. Materials and Methods

### 2.1. Participants

**Figure 1:**
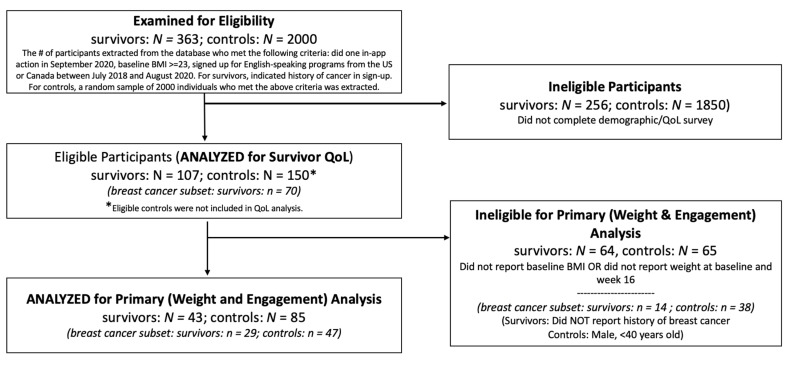
Flow diagram of participant eligibility. *N* refers to the main sample and *n* refers to the subsample of breast cancer survivors only.

Only participants who had already signed up for the program were analyzed in this study. All participants provided consent for their program data to be used for research. Participants were also given the option to opt out. Participants were eligible if they signed up between July 2018 and August 2020, were still on the program (i.e., did one in-app action) in September 2020, had a BMI ≥ 25 kg/m^2^, and had indicated a history of cancer during program sign-up (*N* = 363). A random sample of matched controls who had a similar BMI range (overweight or obese), 0–1 chronic health conditions (e.g., hypertension, type 2 diabetes), signed up for the program during the same time period, and were still on the program were selected (*N* = 2000). These criteria were selected so that controls were matched on key factors that could influence weight loss outcomes. Controls and survivors were contacted by email with a survey invitation at the time of data collection (September 2020). The survey measured self-reported cancer-related QoL (for survivors) and demographics (for survivors and controls). All participants were offered the chance to win one of three $100 gift cards for survey completion. 107 survivors and 150 controls completed the survey and were included in the study (see Figure 1 for a diagram of inclusion). For all participants, self-reported weight, engagement, and physical activity data were extracted from the program database from baseline through week 16 (the minimum length of the core weight loss program).

Because not all participants weighed in every week, weight and engagement analyses included only individuals who reported their weight at baseline and week 16 (43 survivors, 85 controls). Eligible survivors who responded to the survey, even if they did not report their weight at baseline and week 16, were included in descriptive QoL analysis (107 survivors).

In addition to these analyses, we analyzed a subset of breast cancer survivors. From the survivor samples described above, we included any participant who reported a history of breast cancer (*n*= 29 for weight and engagement analyses, *n* = 70 for QoL analyses). For weight and engagement analyses with this subset, controls were matched to breast cancer survivors on gender and age, such that from the original sample of 85 matched controls, only those who were female and ≥40 years old were selected (*n* = 47 controls).

### 2.2. Digital Platform

Noom is a mobile program that has been found to result in clinically significant weight loss in RCTs of a general population with overweight or obesity [14]. It is a publicly available program; individuals who elect to continue with the program after the free trial pay for a subscription. The program is based on cognitive behavioral therapy (CBT) and motivational interviewing techniques, which aid in weight control and increasing motivation to make behavioral changes [15]. Individuals are provided daily articles informed by federal guidelines and empirical work on healthy diet, physical activity, and the psychology of behavior change. The articles about nutrition are informed by MyPlate recommendations as well as empirical work on energy density [16,17]. Noom has a food color system that categorizes foods based on energy density, in terms of high (red), medium (yellow), and low (green) energy density. Previous work has shown that adherence to the food color system is associated with greater weight loss on Noom [18]. Individuals are guided through the entire program with behavior change principles derived from CBT, motivational interviewing, and third-wave CBT (e.g., dialectical behavior therapy) techniques, as well as behavior change techniques like self-monitoring and goal setting [15,19,20]. Individuals are provided with mobile logging features to self-monitor their weight and exercise, as well as a virtual group and the ability to exchange text messages with a 1:1 health coach. The health coach helps the individual to set individualized goals, recognize barriers, and identify individualized solutions to barriers. The coach also discusses awareness of behaviors and barriers (e.g., self-awareness), checks in on progress towards goals, and provides support to users [21]. A group coach oversees the group posts. The program does not have a required length and users can participate for as long as they would like.

### 2.3. Measures

Weight: Participants self-reported their weight on the program. Weight measurements from week 1 through 16 were extracted from the program database. Individuals are encouraged, but not required, to log their weight daily.

Engagement: We gathered data from the weight loss program database to assess differences in physical activity (steps) and program engagement. Steps were tracked by smartphone sensors, wearable devices connected to the program, or manually entered by participants. As in past work, engagement was measured by the number of times per week that participants self-reported their weight or exercises on the program, and the number of times they messaged their coach, which was tracked by the program [22]. Coaches reach out to users at least once a week, and individuals are encouraged, though not required, to log their exercise daily.

Quality of life: Survivors self-reported cancer history and cancer-related QoL via survey. Quality of life questions were adapted from the European Organization for Research and Treatment Core Quality of Life Questionnaire (EORTC QLQ-C30) [23] and transformed into scores ranging from 0 to 100, including overall quality of life rating with higher scores indicating better QoL; functioning scales with higher scores indicating better functioning role functioning, emotional function, cognitive functioning, social functioning; and symptom scales with higher scores indicating more severe symptoms including nausea, pain, dyspnea, insomnia, appetite loss, constipation, and diarrhea.

### 2.4. Statistical Analysis

Analyses were conducted in R (v 3.6.0) with α of 0.05. Descriptive statistics are expressed in means and standard deviations for normally distributed variables or median and interquartile range (IQR) for non-normally distributed variables. Weight loss constituted week 16 weight subtracted from baseline weight. Linear regressions were used to compare survivors and matched controls’ weight loss while accounting for baseline BMI, age, gender, and engagement, since these are all factors that can influence the amount of weight lost [24,25,26]. *T*-tests, Mann-Whitney U test and chi-squared tests compared cancer survivors and matched controls on engagement and demographics. Cancer-related quality of life is presented descriptively with means and standard deviations.

## 3. Results

### 3.1. Demographics

Most cancer survivors had a history of breast cancer (*n* = 70). Other cancer types included melanoma (*n* = 9), cervical (*n* = 6), non-Hodgkin lymphoma (*n* = 5), renal (*n* = 5), skin (non-melanoma; *n* = 5), endometrial (*n* = 4), colon (*n* = 3), leukemia (*n* = 3), ovarian (*n* = 3), bladder (*n* = 2), rectal (*n* = 2), bone (*n* = 1), head and neck (*n* = 1), liver (*n* = 1), lung (*n* = 1), pancreatic (*n* = 1), prostate (*n* = 1), and other (*n* = 13). Survivors could indicate more than one cancer type. A majority of survivors reported having received chemotherapy (IV or pills) (63.5%), radiation (52.3%), and surgery (86.9%). Survivors could report more than one type of treatment. Most survivors received treatment 1 to less than 5 years ago (31.7%) or 5 to less than 10 years ago (24.3%), while 11.2% received treatment 10 or more years ago. For breast cancer survivors only, most reported receiving chemotherapy (IV or pills) (68.6%), radiation (68.6%), and surgery (94.3%). Controls had no history of cancer but had 0–1 chronic conditions. The most common chronic conditions were hypertension (13%) and depression (12%).

Demographic characteristics for all eligible cancer survivors and controls, as well as those included in primary analyses of weight and engagement, are displayed in Table 1. There were significant differences in employment status, where more cancer survivors were retired, and more controls worked 40+ hours per week. Eligible cancer survivors included significantly more females and were significantly older than controls. The same pattern for age but not gender emerged in the subset of breast cancer survivors and matched controls included in primary analyses.

### 3.2. Weight Loss

Participants who had baseline and week 16 weight data were included in the weight loss outcomes (43 survivors, 85 controls). Among this subset, cancer survivors (M = 60.46, SD = 8.82) remained significantly older than matched controls (M = 47.51, SD = 13.07; t (115.69) = 6.64, *p* < 0.001) and had differing employment status. As seen in Table 2, matched controls lost significantly more weight (in kg) than cancer survivors [t (90.81) = −2.07, *p* = 0.04], but the percentage of body weight lost did not differ significantly. After controlling for gender, age, engagement, and baseline BMI, controls lost 1.9 kg more than cancer survivors (B = −1.90, S.E. = 0.95, *p* = 0.05). Overall, males lost more weight than females (B = −3.45, S.E. = 1.00, *p* < 0.001) and as participants aged, they lost more weight (B = −0.09, S.E. = 0.03, *p* = 0.007). The more participants engaged, the more weight they lost (B = −0.54, S.E. = 0.13, *p* < 0.001). Results did not change when controlling for engagement status, which was significantly different across groups but did not emerge as a significant predictor of weight (all *p*s > 0.60).

### 3.3. Engagement

There were differences between the groups in weekly engagement (Table 2). Cancer survivors sent more messages to their coaches compared to controls [W = 605862, *p* < 0.001]. However, controls logged more exercise sessions [W = 432425, *p* < 0.001], and took more steps [W = 415962, *p* < 0.001] compared to cancer survivors.

### 3.4. Quality of Life

Global QoL for all eligible survivors (*N* = 107) at the time surveys were administered was 72.0 on average (SD = 18.5). Scores on the functional subscales were as follows: role functioning: 78.3 (SD = 26.6), emotional functioning: 67.6 (SD = 22.1), cognitive functioning: 80.7 (SD = 19.3), and social functioning: 81.6 (SD = 25.5).

Average symptom scores were as follows: nausea: 4.4 (SD = 10.6), pain: 33.0 (SD = 29.0), dyspnoea: 11.8 (SD = 17.9), insomnia: 40.2 (SD = 28.5), appetite loss: 5.9 (SD = 15.7), constipation: 17.8 (SD = 26.8), diarrhoea: 11.5 (SD = 21.5), financial difficulties: 14.3 (SD = 25.9).

### 3.5. Subset Analysis

Due to the large proportion of breast cancer survivors in our sample, we conducted a subset analysis to compare breast cancer survivors to the controls. Compared to controls, breast cancer survivors wrote more coach messages (breast cancer survivors: Median = 2, IQR = 1–; controls: Median = 1, IQR = 0–3; *p* = 0.001), and logged fewer instances of exercise (breast cancer survivors: Median = 0, IRQ = 0–3; controls: Median = 1, IQR = 0–6; *p* < 0.001) and steps (breast cancer survivors: Median = 19996, IQR = 10181–38656; controls: Median = 33367, IQR = 12481–49324; *p* < 0.001). They logged their weight similarly to controls (breast cancer survivors: Median = 7, IQR = 5–7; controls: Median = 7, IQR = 5–7). When controlling for age, baseline BMI, and a composite score of overall engagement, breast cancer survivors lost significantly less weight than controls (B = −2.07, S.E. = −0.9, *p* = 0.03). Breast cancer survivors lost 5.37kg (SD = 4.39) on average, which was 6.5% body weight loss (SD = 5.4%). Controls lost 7.58kg (SD = 4.38) on average, which constituted 7.2% body weight loss (SD = 4.6%). Eligible breast cancer survivors had a global QoL of 74.5 (SD = 16.0), role functioning of 84.8 (SD = 21.6), emotional functioning of 67.4 (SD = 21.5), cognitive functioning of 80.9 (SD = 19.7), and social functioning of 87.4 (SD = 20.5). Their average symptom scores were as follows: nausea: 3.1 (SD = 8.2), pain: 30.5 (SD = 26.3), dyspnoea: 9.5 (SD = 18.1), insomnia: 40.9 (29.0), appetite loss: 5.2 (SD = 16.7), constipation: 13.8 (SD = 22.3), diarrhoea: 9.0 (SD = 14.9), financial difficulties: 13.8 (SD = 25.7).

## 4. Discussion

This retrospective study examined weight loss and engagement in cancer survivors compared to matched controls who were all trying to lose weight on a digital commercial weight loss program. For this population, real-world weight and engagement outcomes are unknown. This is a particularly pressing question for cancer survivors, who face post-treatment physical and mental health limitations which could impact their engagement and weight loss. It is therefore important to understand how weight loss outcomes for cancer survivors who signed up for a general weight loss program compare to those who do not have a history of cancer. Notably, previous investigations have only taken place in research study settings in which, at the very least, minimal participation requirements were salient. In McCarroll et al. [27], for example, participants were informed that they should provide baseline and follow-up measurements, received training on how to use the commercial program, and were contacted if they did not log food or exercise for more than 3 days in a row. To our knowledge, this is the first study to assess weight loss and engagement in a naturalistic environment where cancer survivors were using the program on their own initiative, without being reminded of participation requirements. We found that cancer survivors lost less weight by 16 weeks than matched controls, but still showed clinically significant weight loss (−5.3 kg or 6.2% body weight). In addition, cancer survivors had lower engagement for self-reported weight and exercise and objectively recorded steps throughout the program. However, when compared with the controls, the cancer survivors sent more messages to their coaches. When the analysis was limited to breast cancer survivors alone, this pattern still held. With regard to weight assessment, this behavior was similar in frequency between breast cancer survivors and controls.

In RCTs of digital commercial programs, cancer survivors lost on average 1.71 kg after 6 months and 2.3 kg after 4 weeks [27,28]. A systematic review found that body weight loss ranged from 2.4 to 6.8% in high-quality RCTs of generalized weight management interventions for survivors [29]. A systematic review of non-commercial weight loss interventions for breast cancer survivors found that survivors lost clinically significant amounts of weight (≥5%) in 14 out of 15 studies [30]. In the context of past studies, our results suggest that cancer survivors with overweight or obesity can lose significant and comparable weight on a digital commercial program, though they do not attain as much weight loss as individuals without a history of cancer.

We found that survivors showed less engagement in terms of logging or physical activity. This corroborates past studies showing that cancer survivors’ engagement is relatively low in digital interventions, as well as work showing that cancer survivors experience fatigue and cognitive barriers to engaging as much as they would like [31,32,33]. We found for the first time to our knowledge that cancer survivors messaged their coaches more than matched controls. This could be because health coaches can provide additional motivation and trust [34]. Future studies should confirm that cancer survivors would benefit from amplified support from health coaches on a digital commercial program.

The study’s additional aim was to describe survivors’ cancer-related QoL at one time point during the program. One time point was chosen to minimize salient study requirements. These descriptive statistics provide rare data on survivors’ QoL after real-world use of a self-managed commercial program and could inform future prospective trials, which are needed to directly compare QoL outcomes. Average global QoL for all survivors was 72.0 (SD = 18.5). In controlled trials of weight management interventions, survivors’ scores were as follows: 56.4 (SD = n.a.) after a 12-week online weight loss intervention, 73.3 (S.E. = 3.7) after a 12-week stage-matched diet and exercise intervention, 79.5 (SD = 18.4) after a 12 week diet and exercise intervention, and 71.4 (SD = 18.8) after a 16-week physical activity and behavior change intervention [35,36,37,38]. A direct comparison cannot be made because of the difference between controlled and digital self-managed settings. Also, the study populations could have different demographic characteristics, with potentially higher socioeconomic status in a commercial program compared to other populations. Therefore, an aim of future work is to compare differences between QoL between cancer survivors and individuals without a history of cancer before and after using Noom. Future work should also compare long-term weight loss between survivors and controls on this type of program.

The study has a few limitations. In order to maximize ecological validity, quality of life was measured once rather than prospectively, weight loss was only analyzed with a subset of participants who provided baseline and 16-week weight measurements, and retrospective analyses were conducted. Because quality of life was not measured at baseline, it is unknown to what extent quality of life improved in cancer survivors over the course of the program. In addition, messaging, physical activity, and weight data were recorded throughout the program, which reduces recall bias, but causal interpretations cannot be made from a retrospective design. Another limitation is that the main outcome was self-reported weight, which can be prone to error or bias [39], and due to the study design, we could not assess its reliability compared to objective measurements. However, it should also be noted that self-reported weight can still be fairly accurate, and this type of observational design could decrease the opportunity for bias that stems from reporting weight directly to researchers (e.g., social desirability bias or from researchers’ expectations) [40,41,42,43,44]. Still, future work should assess the reliability and validity of self-reported weight, and use other objective measurements (e.g., bioimpedance, plethysmography, or bone density measurement). Future research should also use accelerometers or other devices to objectively measure physical activity and calorie consumption. BMI also poses limitations. For instance, BMI does not account for weight variation due to changes in muscle mass (e.g., muscle mass loss from chemotherapy). Future studies should assess body composition specifically. Further, in addition to types of treatment, future work should also consider the duration of cancer treatments. Finally, only participants who did an in-app action in September 2020 were included in the study, since the goal was to investigate outcomes among those who actually participated in the program. This may limit generalizability of the findings and may represent a motivated sample that continued with the program and did not drop out early on.

## 5. Conclusions

This study contributes new knowledge with regard to the use of commercially available digital weight loss programs by cancer survivors outside the context of a clinical trial. Our findings highlight key differences in the experience of cancer survivors versus individuals without a history of cancer. While weight loss and engagement were lower in cancer survivors versus controls, cancer survivors interacted with coaches more than matched controls and lost a clinically significant amount of weight (>5%). Cancer-related QoL was also qualitatively comparable to previous post-weight loss intervention findings. Our results suggest that though cancer survivors can lose significant weight at 16 weeks on a digital commercial program, they may benefit from additional tailoring to improve their weight and engagement. Specifically, survivors may need cancer-specific support in terms of weight loss and motivation to engage in weight management behaviors. This could be done through support from health coaches, as survivors used this resource more than individuals with no history of cancer. These findings support future studies investigating the implementation of digital weight management platforms in oncology care.

## Figures and Tables

**Table 1:** Demographic characteristics of eligible participants.

	All Eligible Participants			Participants Included in Primary Analyses		
	Cancer Survivors (*N* = 107), *N* (%) or Median (IQR)	Matched Controls (*N* = 150), *N* (%) or Median (IQR)	*p*-Value	Cancer Survivors (*N* = 43), *N* (%) or Median (IQR)	Matched Controls (*N* = 85), *N* (%) or Median (IQR)	*p*-Value
**Hispanic/Latino**			1			1
Yes	5 (4.7%)	7 (4.7%)		2 (4.7%)	3 (3.5%)	
No	102 (95.3%)	143 (95.3%)		41 (95.3%)	82 (96.5%)	
**Race**			0.10			0.20
Black or African American	4 (3.7%)	3 (2%)		2 (4.7%)	2 (2.4%)	
White	97 (90.7%)	141 (94%)		39 (90.7%)	81 (95.3%)	
Asian	0 (0%)	4 (2.7%)		0 (0%)	2 (2.4%)	
Other	6 (5.6%)	2 (1.4%)		2 (4.6%)	0 (0%)	
**Employment status**			<0.001			<0.001
Employed, 1–39 h per week	16 (15.0%)	43 (28.7%)		6 (14.0%)	25 (29.4%)	
Employed, 40+ hours per week	36 (33.6%)	84 (56%)		11 (25.6%)	50 (58.8%)	
Not employed	8 (7.4%)	9 (6.1%)		3 (7%)	4 (4.7%)	
Retired	36 (33.6%)	14 (9.4%)		17 (39.5%)	6 (7.1%)	
Disabled, not able to work	11 (10.3%)	0 (0%)		6 (14.0%)	0 (0%)	
**Highest Education**			0.95			0.85
High school degree or some high school	6 (5.6%)	7 (4.7%)		1 (2.3%)	4 (4.7%)	
Some college or vocational training	10 (9.3%)	20 (12.7%)		3 (7.0%)	16.4 (15.9%)	
2-year college degree	12 (11.2%)	12 (8%)		5 (11.6%)	6 (7.1%)	
4-year college degree	38 (35.5%)	48 (32%)		16 (37.2%)	30 (35.3%)	
Some graduate school	8 (7.5%)	13 (8.7%)		3 (7.0%)	7 (8.2%)	
Graduate degree	32 (29.9%)	49 (32.6%)		15 (34.8%)	23 (27.1%)	
I prefer not to answer	1 (0.9%)	1 (0.7%)		0 (0%)	1 (1.2%)	
**Gender**			<0.001			0.20
Female	100 (93.5%)	114 (76%)		39 (90.7%)	67 (78.8%)	
Male	7 (6.5%)	35 (23.3%)		4 (9.3%)	17 (20%)	
Other	0 (0%)	1 (0.7%)		0 (0%)	1 (1.2%)	
Current Age *	61 (53–67)	49 (38–58)	<0.001	62 (53.5–66.5)	49 (38–58)	<0.001

Note. * denotes variables that deviated from a normal distribution, so independent 2-group Mann-Whitney U tests were employed. Chi-squared tests were used for all other variables.

**Table 2:** Weight and engagement for cancer survivors and matched controls.

	Cancer Survivors (*N* = 43), Median (IQR) or Mean (SD)	Matched Controls (*N* = 85), Median (IQR) or Mean (SD)	*p*-Value
Baseline BMI *	32.78 (29.15–37.48)	31.82 (28.83–36.79)	0.50
Weight loss (kg)	−4.72 (4.34)	−6.52 (4.77)	0.04
Weight loss (%)	−6.20 (5.18)	−7.39 (4.67)	0.08
Engagement per week			
Coach messages *	2 (0–3)	1 (0–3)	<0.001
Weigh ins *	7 (4–7)	7 (5–7)	0.07
Exercises *	0 (0–2)	1 (0–7)	<0.001
Steps *	20321 (9386–39550)	35034 (16764–51781)	<0.001
Meals logged *	26 (19–33)	26 (21–31)	0.98
Articles read *	25 (11–28)	26 (12–28)	0.07

Note. * denotes variables that deviated from a normal distribution, so independent 2-group Mann-Whitney U tests were employed. *T*-tests were used for all other variables.

## Data Availability

Restrictions apply to the availability of these data. Data was obtained from Noom and are available by request from the corresponding author with the permission of Noom.
